# Development and implementation of an integrated oral health and nutrition education program using community children’s cafeterias: A pilot study

**DOI:** 10.1371/journal.pone.0357410

**Published:** 2026-09-03

**Authors:** Rena Hidaka, Akiko Kojo, Yuji Masuda, Shintaro Hata, Misaki Tanaka, Tona Watanabe, Koichiro Matsuo

**Affiliations:** 1 Department of Oral Health Sciences for Community Welfare, Graduate School of Medical and Dental Sciences, Institute of Science Tokyo, Bunkyo, Tokyo, Japan; 2 Faculty of Food and Nutritional Sciences, Japan Women’s University, Tokyo, Japan; 3 Division of Oral and Maxillofacial Biology, Institute for Oral Science, Matsumoto Dental University, Nagano, Japan; 4 Apple Dental, Tokyo, Japan; University of Petra (UOP), JORDAN

## Abstract

**Background:**

Dental and oral health activities that promote awareness of oral health are essential in children’s nutrition education. We aimed to evaluate the effects of the Comprehensive Awareness Modification of Mouth, Chewing And Meal (CAMCAM) program for kids, in which participants gather monthly to learn about oral health and nutrition while consuming a textured meal at community children’s cafeterias.

**Methods:**

This single-group pre–post pilot intervention without a control group included 52 children and 32 parents/guardians who regularly attended two community children’s cafeterias in Tokyo, Japan. They participated monthly in six sessions in which they were provided a “munchy” textured meal and a 10-minute lecture on chewing, oral health, and food and nutrition. Participants completed questionnaires assessing their awareness of the subject and food intake frequency, before and after the intervention. Changes in awareness and behaviors following participation in the program were analyzed.

**Results:**

The median ages of the children and parents were 8.5 (6–16) and 43.0 (36–50) years, respectively. The frequency of self-observation of the oral cavity among children increased. Daily fluoride use frequency increased from 61.5% to 76.9% (p = 0.020). The number of children who reported not considering nutritional balance decreased from 63.5% to 38.5% (p = 0.022). The number of parents/guardians who actively practiced a nutritionally balanced diet non-significantly increased from 56.2% to 75.0%. Food intake frequencies for meat, dairy products, and tubers, among caregivers, and that for rice, tubers, and fruits among children increased post-intervention. The number of participants who did not consider the number of chewing cycles decreased.

**Conclusion:**

Participation in CAMCAM-P for kids was associated with favorable changes in oral health awareness and attitudes toward nutritional balance among children and parents/guardians. As a pilot study without a control group, these findings should be considered preliminary. Larger controlled studies are warranted to confirm effectiveness.

## Introduction

Oral health habits, food preferences, and dietary behaviors established in early childhood are reported to have long-lasting effects throughout the life course [1–3]. In recent years, children’s dietary challenges have become increasingly complex. These include imbalanced nutrient intake and skipped breakfast, raising concerns about their long-term health impacts such as obesity [4,5]. Furthermore, there is a decrease in the frequency of breakfast consumption and intake of fruits and vegetables during the transition from elementary to junior high school [6], highlighting the importance of establishing desirable dietary habits at an early stage.

In Japan, delays in the development of oral functions in children are a public health concern. To emphasize the importance of early intervention, *oral hypofunction in children* was added to the list of diseases covered by the national dental health insurance system in 2018 [7]. Insufficient development of oral function can result in difficulties in eating and may contribute to imbalanced nutrition. Previous research reported that the prevalence of pediatric oral hypofunction among dental outpatients aged 0–17 years was 2,431.3 per 100,000 children (2.4%), and it is suggested that the actual number of affected children may be even higher [8]. Therefore, oral hypofunction may be one of the factors underlying recent dietary issues among children. Accordingly, collaboration with dental and oral health activities that promote awareness of oral health and chewing is essential when designing nutrition education programs for children.

Parental characteristics also play a significant role. Parents’ sociodemographic characteristics, such as educational level, as well as their oral health behaviors—toothbrushing practices, frequency of dental visits, and oral health education—have been shown to exert both direct and indirect effects on children’s oral health behaviors and status [9–13]. In addition, higher levels of parental nutrition knowledge have been associated with more desirable dietary behaviors in children [14]. These findings indicate that interventions targeting children and their caregivers are necessary to establish healthy habits from early childhood.

The Comprehensive Awareness Modification of Mouth, Chewing, and Meal (CAMCAM) program (CAMCAM-P) was originally developed to prevent frailty among community-dwelling older adults [15–18]. The CAMCAM-P aims to promote behavioral changes related to oral health, diet, and physical health by incorporating educational components on oral health and nutrition while participants consume a specially designed CAMCAM textured meal (*CAMCAM* bento®) that emphasizes chewing. A 6-month intervention has been shown to promote positive changes in awareness related to oral health, nutrition, and chewing among older adults with oral frailty, leading to improvements in oral and physical frailty. In the present study, the CAMCAM-P was adapted for children and their caregivers, to examine whether this modified program could induce changes in awareness and behaviors related to oral health, nutrition, and chewing in both groups.

## Materials and methods

### Participants

This study was designed as a pre–post pilot intervention without a control group and was conducted between April 2023 and December 2024. A total of 84 participants (52 children and 32 parents/guardians) were recruited from two community children’s cafeterias in Tokyo. These cafeterias were selected based on their established collaboration with the research team and their stable user populations, which made them suitable settings for a feasibility-oriented pilot intervention. A convenience sampling approach was used, as the primary aim of this study was to assess the feasibility and preliminary changes associated with the integrated oral health and nutrition education program. Participants were eligible if they were regular attendees of the cafeterias and did not have any food allergies that would interfere with the program activities. Written and verbal informed consent was obtained from parents or legal guardians, and assent was obtained from all participating children. Participation was voluntary, and all data were anonymized prior to analysis.

This study was conducted in accordance with the principles of the Declaration of Helsinki (World Medical Association, 2015) and the Ethical Guidelines for Life Science and Medical Research Involving Human Subjects (March 23, 2021). Ethical approval was obtained from the Ethics Committee of the Faculty of Dentistry, Institute of Science Tokyo (approval number: D2022-026). The study was also registered with the University Hospital Medical Information Network (UMIN) (trial ID: UMIN000048328).

### Intervention

The Comprehensive Awareness Modification of Mouth, Chewing, and Meal (CAMCAM) program for Children (CAMCAM-P for kids) was implemented over a 6-month period. Once a month, participants attended an educational session on oral health, chewing, and nutrition, and received a specially designed bento meal (*CAMCAM Bento*) intended to promote chewing.

Each educational session lasted approximately 10 min. Overall, six sessions were conducted during the intervention period. Lectures were delivered by dentists, dental hygienists, and registered dietitians using presentation slides covering topics related to oral health, chewing, and nutrition.

The *CAMCAM Bento* consisted of rice as the staple food, a main dish, and side dishes. The bento was designed to increase chewing frequency by (1) using foods with higher chewiness, (2) cutting ingredients into larger pieces, (3) shortening cooking times, and (4) reducing moisture content.

In addition, a mobile application was used to disseminate short informational messages (approximately 200 characters) once a week on topics related to oral health, nutrition, and chewing, and to allow participants to view recorded lectures.

### Questionnaire

A child-specific CAMCAM Checklist was developed to evaluate changes in participants’ awareness and behaviors associated with participation in the program. The questionnaire was developed by the research team based on a review of previous studies and existing oral health and nutrition education materials [19–21], and through discussion with dentists and dental hygienists. The checklist was designed as an original instrument tailored to the objectives and context of this study. The full questionnaire is provided in Supplementary Appendix 1 to ensure transparency and reproducibility.

The questionnaire consisted of items related to oral health, dietary behaviors, and chewing.

Oral health items included questions on toothbrushing behaviors (frequency of toothbrushing, frequency of autonomous toothbrushing [children only], and frequency of parental-assisted brushing [parents/guardians only]), frequency of observing the oral cavity, regular dental visits, and frequency of fluoride use.

Diet-related items included dietary habits (number of meals, frequency of breakfast consumption, frequency of eating breakfast together, and consideration of nutritional balance) [20]. Food intake diversity was assessed using the Dietary Variety Score [21], a previously established measure.

Chewing-related items assessed the frequency of behaviors such as “paying attention to the number of chewing cycles,” “leaving hard foods uneaten,” and “consuming foods that require chewing.”

Changes in awareness and behaviors related to oral health, nutritional balance, and chewing were evaluated using the Transtheoretical Model (TTM) [22]. The TTM categorizes behavior change into five stages: precontemplation (not currently performing the behavior and not interested), contemplation (not currently performing the behavior but intending to do so within 6 months), preparation (not currently performing the behavior but intending to do so within 1 month), action (performing the behavior for < 1 month), and maintenance (performing the behavior for ≥ 1 month).

In the original TTM, maintenance is defined as sustaining the behavior for ≥ 6 months; however, Walton, et al. [23] recommended a shorter evaluation period of 2 months for studies involving fifth- and sixth-grade elementary school students, as 6 months exceeds the recall capacity of children in this age group. Given that the participants in the present study included children from the first grade of elementary school and above, who were of an even younger age range, the maintenance stage for children was defined as “sustaining the behavior for ≥ 1 month.”

### Measures

Oral function assessments were conducted only in children. Oral function was evaluated using the Simplified Debris Index and Simplified Calculus Index of the Oral Hygiene Index–Simplified [24], the PMA index [25], tongue–lip motor function (oral diadochokinesis [ODK]), masticatory performance, lip pressure, and tongue motor function. Additionally, lip closure, lingual frenulum abnormalities, mouth breathing, palatine tonsil hypertrophy, and the presence of unfavorable oral habits were evaluated. Interviews were also conducted with the children and their parents or guardians.

Masticatory performance was evaluated using a chewing ability test gum (XYLITOL® chewing ability test gum, Lotte Co., Ltd.). Participants were instructed to chew the gum for 1 min, and the degree of color change was assessed using a five-grade color chart [26–28].

ODK was assessed using an automatic speech analyzer (Kenkou-kun Handy, Takei Scientific Instruments Co., Ltd., Japan). The number of repetitions of monosyllabic sounds (/pa/, /ta/, /ka/) was recorded. Oral motor ability related to articulation was evaluated by measuring the number of syllables produced per second [29].

Lip pressure was measured using a dental lip strength measurement device (Lipple-kun, Shofu Inc., Japan) [30]. Participants were instructed to insert a button into the oral vestibule and close their lips. After pulling the button horizontally, the force required to dislodge the button from the oral vestibule was measured. Following one practice trial, measurements were taken three times, and the mean value was calculated as the lip pressure.

Tongue motor function was assessed using a tongue pressure measurement device (TPM-01, JMS Co., Ltd.). Participants were instructed to press a small balloon-type disposable oral probe against the palate as forcefully as possible [31,32]. After one practice trial, measurements were performed three times, and the mean value was used for analysis.

Based on a comprehensive evaluation of questionnaire responses, measurement results, and interview findings, the prevalence of oral hypofunction was determined [7,33].

### Statistical analysis

Comparisons before and after the intervention of the CAMCAM-P for kids were analyzed using the Wilcoxon signed-rank test. A p value of <0.05 was considered statistically significant. All statistical analyses were performed using SPSS version 29.0 software (SPSS Inc., Chicago, IL, USA).

## Results

The basic characteristics of the 84 participants (52 children and 32 parents/guardians) are shown in Table 1.

**Table 1:** Participant characteristics.

	Children(N =52)	Parents(N= 32)
Mean age (years [SD])	8.5 (2.1)	43.0 (4.9)
Sex (n [%])	Men: 24 (46.2) Female: 28 (53.8)	Men: 2 (6.3) Female: 30 (93.8)
Height (cm)	135.3 (14.7)	159.3 (7.2)
Weight (kg)	28.2 (9.5)	58.7 (15.8)
Rohrer indexBMI (parents) (n [%])	Underweight: 14 (26.9) Normal: 31 (59.6) Obese: 5 (9.6)	Underweight: 2 (6.3) Normal: 23 (71.9) Obese: 7 (21.9)
Age distribution for children (years)	6: 8 (15.4) 7: 14 (26.9) 8: 5 (9.6) 9: 10 (15.4) 10: 8 (15.4) 11: 3 (5.8) 12: 3 (5.8) 16: 1 (1.9)	

BMI, body mass index

The results of the CAMCAM checklist are presented in Tables 2, 3, 4. Regarding items related to oral health, children showed a significant increase in the frequency of observing their oral cavity (p = 0.039). Additionally, the proportion of children who reported using fluoride “every day” increased from 32 (61.5%) to 40 (76.9%). The number of parents/guardians who reported “rarely using fluoride” decreased from 16 (50.0%) to 6 (18.8%). There were no significant changes in the frequency of parental-assisted tooth brushing, the number of daily tooth-brushing episodes, or the frequency of regular dental visits among parents/guardians.

**Table 2:** Items of the CAMCAM Checklist for children.

	Baseline	Final	r	p-value
**Toothbrushing frequency**	1.6 (0.5)	1.6 (0.6)	0.00	1.000
**Brushes teeth voluntarily (children only)**	Almost every day: 24 (46.2) Every 2 days: 3 (5.8) 1–2 times per week: 7 (13.5) Almost never: 18 (34.6)	Almost every day: 29 (55.8) Every 2 days: 7 (13.5) 1–2 times per week: 4 (7.7) Almost never: 12 (23.1)	−0.19	0.170
**Observes oral cavity**	Almost every day: 9 (17.3) Every 2 days: 3 (5.8) 1–2 times per week: 7 (13.5) Almost never: 33 (63.5)	Almost every day: 11 (21.2) Every 2 days: 10 (19.2) 1–2 times per week: 6 (11.5) Almost never: 25 (48.1)	−0.29	0.039
**Regular dental visits**	Yes: 37 (71.6) Unsure: 6 (11.5) No: 9 (17.3)	Yes: 38 (73.1) Unsure: 5 (9.6) No: 9 (17.3)	−0.25	0.858
**Frequency of fluoride use**	2.1 (1.5)	1.7 (1.3)	−0.32	0.020
**Number of meals**	Two: 2 (3.8) Three: 48 (92.3) Over four: 2 (3.8)	Two: 2 (3.8) Three: 47 (90.4) Over four: 3 (5.8)	−0.04	0.763
**Eats breakfast**	Almost every day: 51 (98.1) 1–2 times per week:1 (1.9)	Almost every day: 50 (96.2) Every 2 days: 2 (3.8)	0.00	1.000
**Eats breakfast together with family**	Almost every day: 44 (84.6) Every 2 days:1 (1.9) 1–2 times per week: 4 (7.7) Almost never: 3 (5.8)	Almost every day: 42 (80.8) Every 2 days: 2 (3.8) 1–2 times per week: 3 (5.8) Almost never: 5 (9.6)	−0.20	0.157
**Considers nutritional balance during meals**	Almost every day: 7 (13.5) Every 2 days: 4 (7.7) 1–2 times per week: 8 (15.4) Almost never: 33 (63.5)	Almost every day: 10 (19.2) Every 2 days: 8 (15.4) 1–2 times per week: 14 (26.9) Almost never: 20 (38.5)	−0.32	0.022
**Pay attention to number of chews**	Yes: 9 (17.3) Unsure: 10 (19.2) No: 33 (63.5)	Yes: 3 (5.8) Unsure: 5 (9.6) No: 44 (84.6)	−0.34	0.015
**Leaves hard foods uneaten (1: always, 5: never)**	2.4 (0.9)	2.4 (0.9)	0.00	1.000
**Eats foods that require chewing**	Almost every day: 19 (36.5) Every 2 days: 6 (11.5) 1–2 times per week: 14 (26.9) Almost never: 13 (25.0)	Almost every day: 22 (42.3) Every 2 days: 12 (23.1) 1–2 times per week: 8 (15.4) Almost never: 10 (19.2)	−0.24	0.085

**Table 3:** Items of the CAMCAM Checklist for parents/guardians.

	Baseline	Final	r	p-value
**Toothbrushing frequency**	2.2 (0.6)	2.2 (0.6)	−0.04	0.803
**Parent-supervised brushing (parents only)**	Almost every day: 14 (43.8) Every 2 days: 4 (12.5) 1–2 times per week: 1 (3.1) Almost never: 13 (40.6)	Almost every day: 15 (48.4) Every 2 days: 1 (3.1) 1–2 times per week: 5 (15.6) Almost never: 10 (31.3)	−0.06	0.722
**Observes oral cavity**	Almost every day: 6 (18.8) Every 2 days: 3 (9.4) 1–2 times per week: 11 (34.3) Almost never: 12 (37.5)	Almost every day: 8 (25.0) Every 2 days: 4 (12.5) 1–2 times per week: 10 (31.3) Almost never: 10 (31.3)	−0.20	0.250
**Regular dental visits**	Yes: 20 (62.5) Unsure: 6 (18.8) No: 6 (18.8)	Yes: 22 (68.8) Unsure: 5 (15.6) No: 5 (15.6)	−0.08	0.660
**Frequency of fluoride use**	2.6 (1.6)	2.0 (1.5)	−0.41	0.021
**Number of meals**	Two:1 (3.1) Three: 31 (96.9)	Two: 6 (18.8) Three: 26 (81.3)	−0.40	0.025
**Eats breakfast**	Almost every day: 27 (84.4) Every 2 days: 1 (3.1) 1–2 times per week: 3 (9.4) Almost never:1 (3.1)	Almost every day: 27 (84.4)1–2 times per week: 3 (9.4) Almost never: 2 (6.3)	−0.07	0.683
**Eats breakfast together with family**	Almost every day:13 (40.6) Every 2 days:2 (6.3) 1–2 times per week:8 (25.0) Almost never: 9 (28.1)	Almost every day: 15 (46.9) Every 2 days: 2 (6.3) 1–2 times per week: 5 (15.6) Almost never: 10 (31.3)	−0.09	0.958
**Considers nutritional balance during meals**	Almost every day: 18 (56.3) Every 2 days: 5 (15.6) 1–2 times per week: 5 (15.6) Almost never: 4 (12.5)	Almost every day: 20 (62.5) Every 2 days: 4 (12.5) 1–2 times per week: 5 (15.6) Almost never: 3 (9.4)	−0.10	0.555
**Pays attention to number of chews**	Yes: 9 (28.1) Unsure: 11 (34.4) No: 12 (37.5)	Yes: 12 (37.5) Unsure: 2 (6.3) No: 18 (56.3)	−0.02	0.923
**Leaves hard foods uneaten (1: always, 5: never)**	2.9 (1.3)	2.7 (0.7)	−0.10	0.564
**Eats foods that require chewing**	Almost every day: 10 (31.3) Every 2 days: 4 (12.5) 1–2 times per week: 4 (12.5) Almost never: 14 (43.8)	Almost every day: 13 (40.6) Every 2 days: 7 (21.9) 1–2 times per week: 5 (15.6) Almost never: 7 (21.9)	−0.28	0.112

**Table 4:** Food intake frequency (1: Almost every day, 2: Every 2 days, 3: 1–2 times per week, 4: Rarely/Almost never).

	Children (N = 52)				Parents (N = 32)			
	Baseline	Final	r	p value	Baseline	Final	r	p value
**Rice**	1.2 (0.4)	1.0 (0.1)	−0.35	0.011	1.4 (0.7)	1.4 (0.9)	−0.06	0.739
**Bread**	1.9 (1.0)	2.2 (1.1)	−0.08	0.590	2.2 (1.1)	2.3 (1.1)	−0.11	0.520
**Meat**	1.9 (0.9)	1.9 (0.8)	−0.01	0.946	1.7 (0.8)	1.4 (0.6)	−0.41	0.021
**Fish**	2.7 (0.7)	0.7 (0.8)	−0.03	0.837	2.6 (0.7)	2.6 (0.8)	−0.04	0.808
**Eggs**	2.4 (1.0)	2.1 (1.0)	−0.26	0.061	2.0 (0.9)	2.0 (1.0)	−0.01	0.961
**Milk and dairy products**	1.3 (0.7)	1.3 (0.6)	0.00	1.000	1.8 (1.0)	1.4 (0.8)	−0.39	0.028
**Potatoes**	2.6 (0.8)	2.3 (0.8)	−0.30	0.032	2.9 (0.5)	2.4 (0.8)	−0.53	0.003
**Beans**	2.5 (0.9)	2.4 (1.1)	−0.08	0.565	2.4 (0.9)	2.2 (0.8)	−0.22	0.216
**Vegetables**	1.8 (1.0)	1.6 (0.9)	−0.14	0.305	1.3 (0.6)	1.2 (0.5)	−0.14	0.417
**Seaweeds**	2.7 (1.0)	2.5 (0.9)	−0.11	0.414	2.7 (0.7)	2.5 (1.0)	−0.25	0.152
**Fried foods**	2.8 (0.6)	2.7 (0.7)	−0.15	0.286	2.9 (0.8)	2.7 (0.7)	−0.22	0.204
**Fruits**	2.5 (1.1)	2.2 (1.1)	−0.29	0.036	2.5 (0.9)	2.1 (1.0)	−0.22	0.205
**Sweets**	1.3 (0.6)	1.3 (0.6)	−0.02	0.914	1.8 (1.0)	1.7 (1.0)	−0.10	0.560

For nutrition-related items, the number of children who reported that they had not considered dietary balance decreased from 33 (63.5%) before the intervention to 20 (38.4%) after the intervention (p = 0.015). Furthermore, among children, the intake frequency of rice, potatoes, and fruits increased significantly (p = 0.011, 0.032, and 0.036, respectively). Similarly, the intake frequency of meat, eggs, and potatoes increased significantly among parents/guardians (p = 0.021, 0.028, and 0.003, respectively).

Regarding mastication-related items, the frequency of “consuming foods that require chewing” also tended to increase, albeit non-significantly, in children and parents/guardians (p = 0.085 and p = 0.112, respectively).

**Fig 1:**
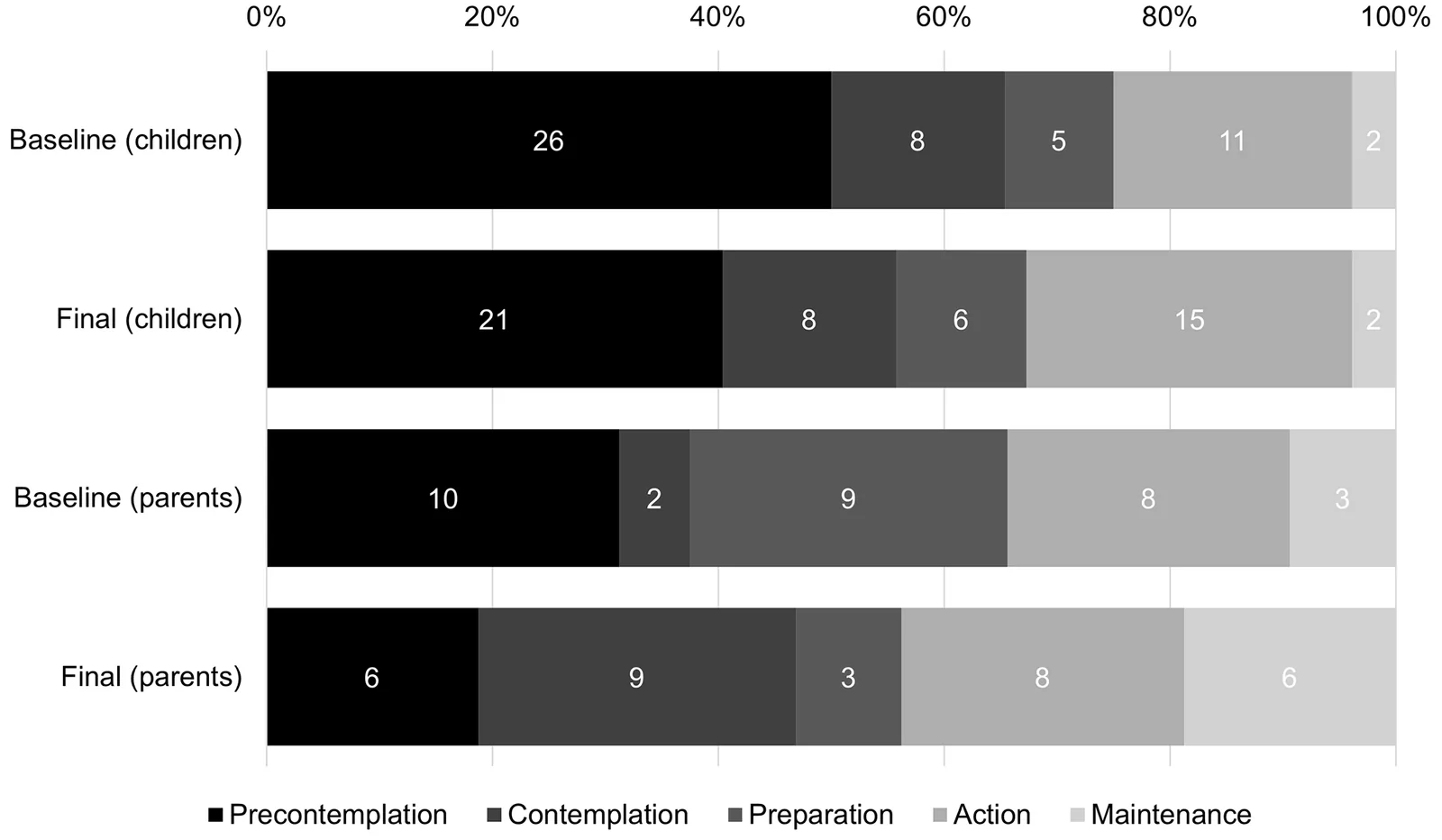
TTM stages for oral health. Distribution of stages of change in children and parents at baseline and at the final assessment.

**Fig 2:**
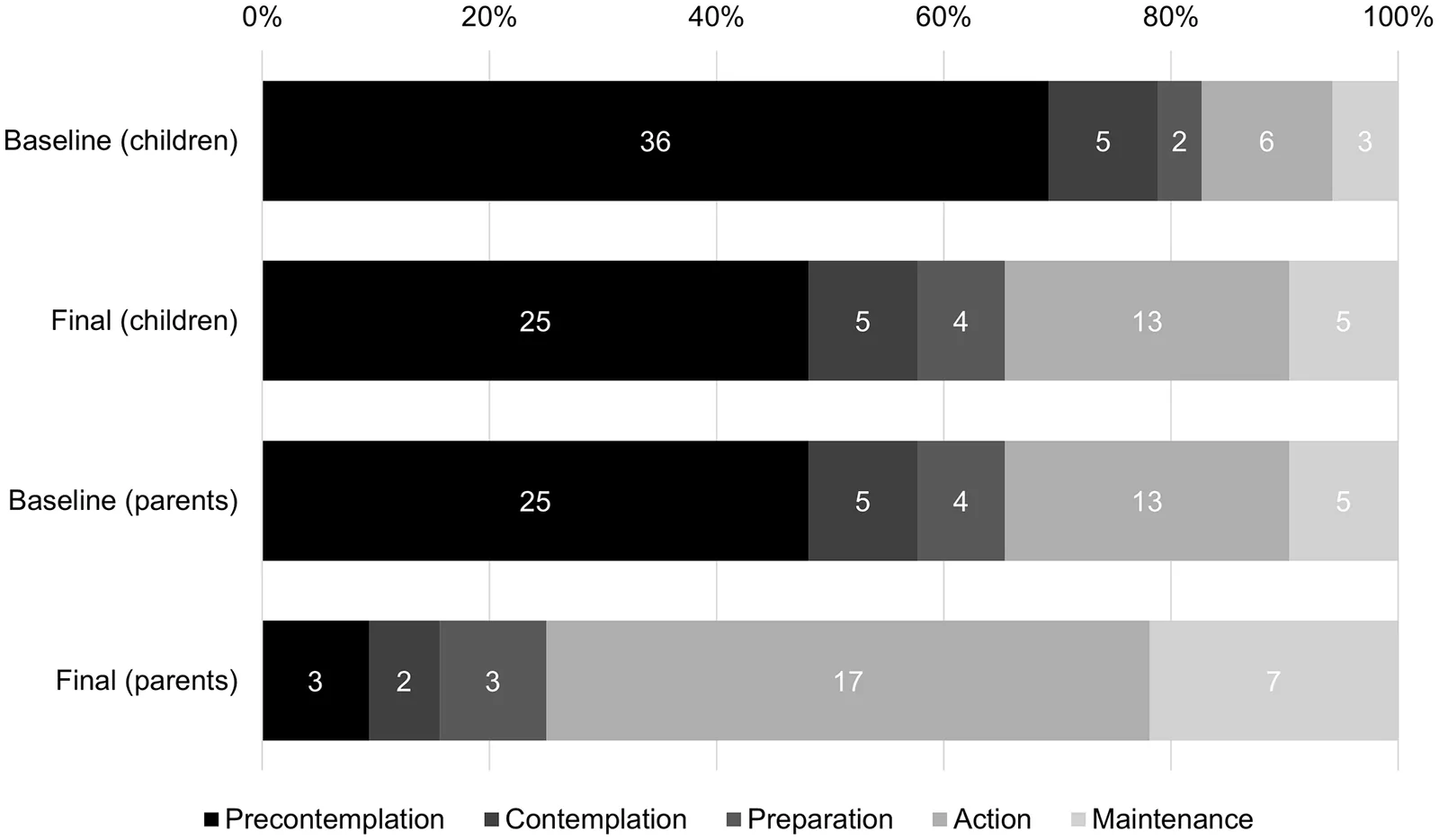
TTM stages for nutritional. Distribution of stages of change in children and parents at baseline and at the final assessment.

**Fig 3:**
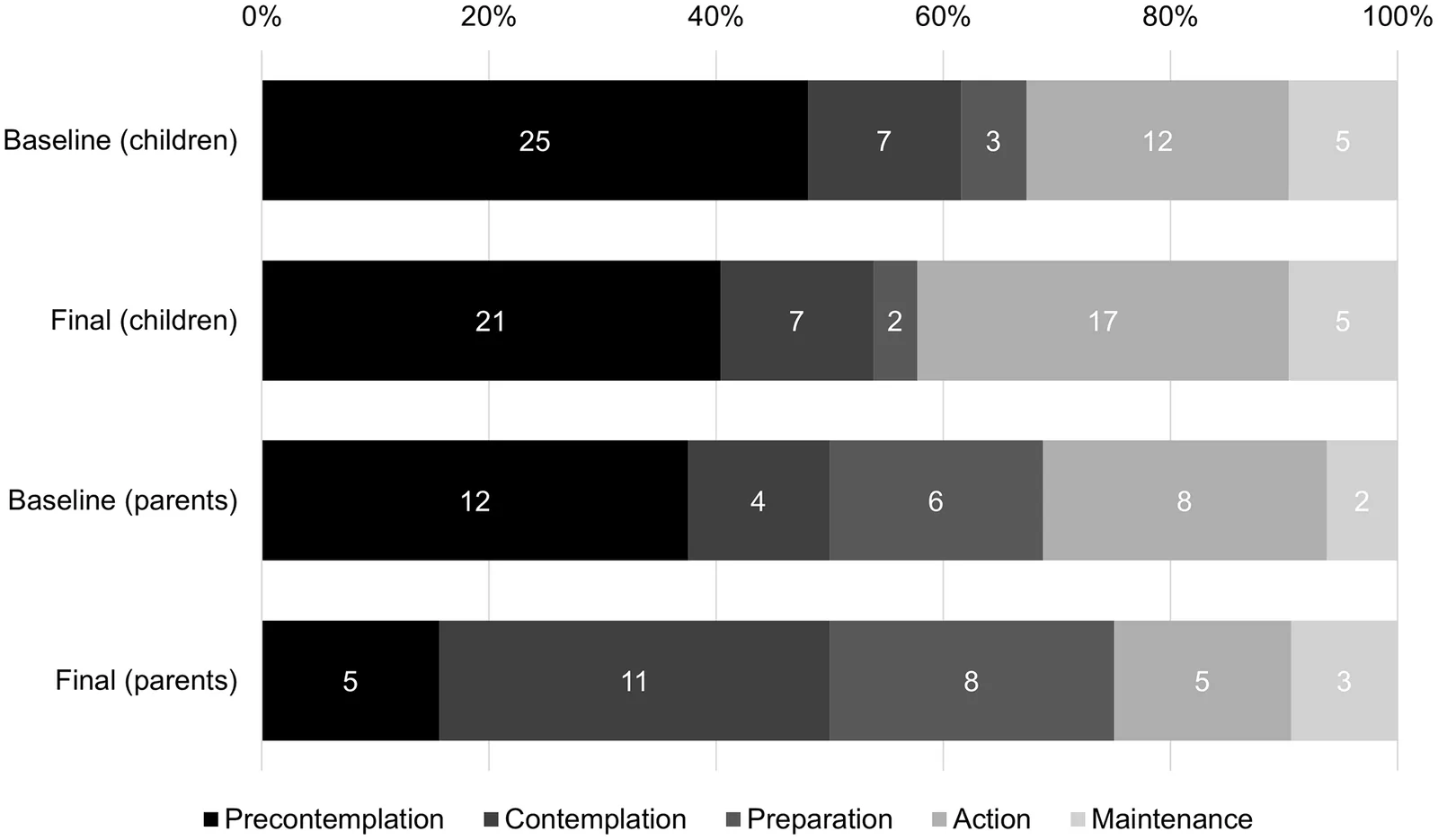
TTM stages for chewing. Distribution of stages of change in children and parents at baseline and at the final assessment.

The results of the behavioral change indicators are shown in Figs 1–3. For oral health behaviors, the proportion of children in the action and maintenance stages increased from 13 (25.0%) to 17 (32.7%) (r = −0.13, p = 0.346), whereas little change was observed among parents/guardians. For nutritional balance, the proportion of participants in the action and maintenance stages increased from 9 (17.3%) to 18 (34.6%) and from 18 (56.2%) to 24 (75.0%), in children and their parents/guardians, respectively (r = −0.39, p = 0.005 and r = 0.09, p = 0.619). Regarding mastication, the proportion of children in the action and maintenance stages increased from 17 (32.7%) to 22 (42.3%). Meanwhile, the proportion of parents/guardians in the precontemplation stage decreased from 12 (37.5%) to 5 (15.6%) (r = −0.18, p = 0.197 and r = −0.13, p = 0.480, respectively).

The results of the oral examinations stratified by school grade are shown in Table 5. In the lower-grade group, significant improvements were observed in ODK /pa/, ODK /ka/, masticatory performance, and tongue pressure (p = 0.014, 0.029, 0.047, and 0.023, respectively). In the higher-grade group, gingival inflammation of the interdental papillae improved significantly (p = 0.010). The prevalence of oral hypofunction was 23.3% and 40.9% in the lower- and higher-grade groups, respectively at baseline, and 36.7% and 13.6% in the lower- and higher-grade groups, respectively, at follow-up (p = 0.096). The increase in the number of children classified as “having oral hypofunction” at follow-up was attributed to an increased number of lower-grade children who reported lip incompetence in the questionnaire, which rose from 6 (20.0%) to 14 (46.7%) (p = 0.046).

**Table 5:** Oral function measurement results.

	Lower grades (N = 30)				Upper grades (N = 22)			
	Baseline	Final	R	p value	Baseline	Final	r	p value
**DI (Debris Index)**	0.9 (0.8)	0.7 (0.6)	−0.18	0.337	1.1 (0.7)	0.8 (0.8)	−0.27	0.210
**CI (Calculus Index)**	0.3 (0.5)	0.2 (0.4)	−0.13	0.480	0.5 (0.6)	0.2 (0.8)	−0.28	0.197
**PMA (Interdental papilla)**	2.9 (2.1)	2.6 (3.0)	−0.09	0.621	5.2 (2.9)	2.6 (3.0)	−0.55	0.010
**PMA (Marginal gingiva)**	1.1 (1.6)	1.8 (3.2)	−0.17	0.350	1.4 (1.9)	2.1 (3.4)	−0.14	0.503
**PMA (Attached gingiva)**	0.1 (2.5)	0.3 (1.2)	−0.14	0.461	0.1 (1.9)	0.4 (1.4)	−0.30	0.157
**PMA total (0–34)**	4.1 (3.4)	4.7 (6.2)	−0.05	0.775	6.8 (4.0)	5.1 (6.5)	−0.34	0.112
**ODK /pa/**	4.1 (1.1)	4.6 (1.0)	−0.45	0.014	5.0 (1.3)	5.5 (0.8)	−0.31	0.148
**ODK /ta/**	4.7 (1.2)	4.6 (1.0)	−0.29	0.111	5.6 (1.5)	5.9 (1.2)	−0.16	0.443
**ODK /ka/**	4.6 (1.1)	4.9 (0.7)	−0.40	0.029	5.4 (1.4)	5.6 (1.0)	−0.16	0.453
**Chewing gum performance**	6.3 (1.3)	7.1 (1.2)	−0.36	0.047	6.3 (1.4)	6.4 (1.9)	−0.15	0.494
**Lip pressure**	9.5 (5.0)	9.3 (3.0)	−0.24	0.192	8.9 (4.3)	9.8 (5.0)	−0.08	0.717
**Tongue pressure**	26.8 (15.0)	32.0 (12.6)	−0.42	0.023	37.1 (8.5)	37.2 (8.2)	−0.02	0.918
**Incomplete lip closure (n)**	6 (20.0)	14 (46.7)	−0.37	0.046	12 (54.5)	8 (36.4)	−0.25	0.248
**Presence of short lingual frenulum (n)**	0 (0)	0 (0)	0.00	1.000	2 (9.1)	0 (0)	−0.30	0.157
**Mouth breathing (n)**	7 (23.3)	9 (30.0)	−0.20	0.285	8 (36.4)	6 (27.3)	0.00	1.000
**Enlarged palatine tonsils (n)**	0 (0)	0 (0)	−0.26	0.157	1 (4.5)	0 (0)	−0.21	0.317
**Oral habit disorder (n)**	9 (30.0)	7 (23.3)	−0.25	0.527	5 (22.7)	2 (9.1)	−0.29	0.180

## Discussion

In this study, a combined oral health and food education program was implemented for children who used a community children’s cafeteria and their parents/guardians, and its effects on oral health, nutrition, mastication, and oral behaviors were evaluated. The program promoted positive changes in oral health behaviors, nutritional balance, and awareness and behaviors related to mastication in both children and parents/guardians.

### 1. Effects on Oral Health, Nutrition, and Mastication

Regarding oral health, increases in the frequency of fluoride use, the proportion of children who brushed their teeth independently, and the proportion of children who observed their oral cavity were observed. These changes may be attributed to the educational sessions, which included instruction on the proper use of fluoride. These likely enhanced participants’ interest in their oral health, leading to immediate changes in feasible oral health behaviors. More than half of the participants already exhibited desirable habits regarding the frequency of tooth brushing and regular dental visits before the intervention. The proportion of participants who were receiving regular dental checkups was higher than the national average [34], suggesting that a ceiling effect may have limited the detection of further changes in these behaviors.

The results of the oral examinations revealed grade-specific characteristics. Among lower-grade children, improvements in lip function, tongue function, and masticatory-related functions were observed. Previous studies targeting Japanese children in the third grade and above have reported sex differences in ODK values depending on grade, possibly influenced by physical growth and development [35]; however, such sex differences were not observed in the present study. In contrast, an improvement in gingival inflammation was noted among higher-grade children. This finding may be explained by the increase in the proportion of higher-grade children who reported brushing their teeth independently, suggesting that heightened awareness of their own oral health contributed to the improvement in gingival status.

At the same time, the number of children classified as having oral hypofunction increased after the intervention. In this study, oral hypofunction was assessed using multiple domains that included both objective functional measures and observational/self-reported indicators, such as lip incompetence. The post-intervention increase in classification was primarily attributable to increased reporting of lip incompetence rather than decline in objective oral functional performance.

By contrast, several objective indicators demonstrated favorable changes after the intervention, including ODK, tongue pressure, and mastication-related measures. Therefore, the apparent inconsistency between improved functional measures and increased oral hypofunction classification may reflect differences in measurement characteristics between subjective indicators and objective performance-based assessments. Participation in the program may have increased awareness of oral habits and symptoms among children and parents/guardians, resulting in more accurate recognition and reporting after the intervention.

Given the exploratory nature of this pilot study and the absence of a control group, these findings should be interpreted cautiously. Further studies with larger sample sizes, validated pediatric assessment criteria, and longitudinal or controlled designs are warranted to clarify the reproducibility and clinical relevance of oral hypofunction findings in children.

There were no significant changes in masticatory performance before and after the intervention. This may be partly due to a temporary decline associated with the transition from deciduous molars to permanent teeth [36]. Nevertheless, masticatory performance is associated with ODK and tongue pressure [37], and the observed improvements in these functions suggest that increased awareness of chewing and behavioral changes related to mastication may have positively influenced oral motor function.

In terms of nutritional balance, both children and parents/guardians demonstrated changes in awareness and behavior. Previous research has shown that parental knowledge of nutritional balance is strongly correlated with children’s fruit and vegetable intake [14]. Accordingly, it is likely that improvements in nutritional knowledge through the program contributed to increased intake frequencies of various food groups. These findings further support the need for implementing interventions in both children and their parents/guardians to improve dietary diversity in children. Additionally, most participants in this study reported eating breakfast regularly. Children who consume breakfast tend to have diets of higher nutritional quality [38] and are more likely to have earlier sleep habits [39], suggesting the establishment of generally favorable lifestyle behaviors. Thus, the study population may have already maintained relatively well-regulated daily habits.

With respect to mastication, the behavioral change indicators demonstrated enhanced awareness and behavioral modification in children and their parents/guardians. Similar improvements in awareness of chewing have been reported in the original version of this program [15]. These findings indicate that the present program is effective in promoting awareness of mastication even when the target population includes children and their parents/guardians, highlighting the robustness and adaptability of the intervention.

### 2. Behavioral change indicators

The TTM comprises four elements: stages of change, processes of change, decisional balance, and self-efficacy. In this study, the stages of change were positioned as a core component of the TTM [40]. Participants’ current status was assessed based on the five stages of change, and interventions were designed to correspond to their respective stages. In this study, the CAMCAM checklist served as the primary outcome measure, while the TTM stages of change were used as secondary outcome indicators to evaluate the effectiveness of the intervention program. Although an upward trend in stage progression was observed in children and their parents/guardians, significant behavioral changes were not observed in most items of the primary outcome. This may be explained by the fact that, at early stages of change, participants’ intention to modify behavior is present but requires additional effort to translate intention into action. Understanding participants’ stages before the intervention suggests that more individualized approaches may have been necessary for those in the early stages. Furthermore, differences in intervention effects between lower- and higher-grade children were suggested, highlighting the potential need for grade-specific program implementation.

### 3. Limitations and future directions

This program combined three elements—oral health, nutrition, and mastication—into a single intervention. Although numerous health programs targeting children exist, including those focused on oral health or food education, most are conducted independently, and few programs integrate these elements into a comprehensive approach. Moreover, interventions are often delivered separately to children and parents/guardians, with limited programs providing the same intervention to both groups. Therefore, if the CAMCAM-P for kids can be disseminated at the community level, it may be effective in supporting the early development of oral functions and the establishment of desirable dietary habits.

This study has some limitations. First, this was a pilot study using a single-group pre–post design without a contemporaneous control group; therefore, causal inferences cannot be made, and the observed changes may have been influenced partly by normal growth and development. Second, the sample size was relatively small, which limited subgroup analyses. Third, participants ranged widely in age and developmental stage, from early elementary school children to adolescents, and responses to the intervention may have varied according to school grade and behavioral readiness stage. Future studies with larger samples and controlled designs are warranted to confirm these preliminary findings and further refine the CAMCAM-P for kids by optimizing intervention frequency according to behavioral stage and tailoring program content to developmental level.

## Conclusion

This preliminary study demonstrated that the CAMCAM-P for kids, which integrates oral health education into food education, may promote improvements in oral health behaviors and awareness of nutritional balance in children and their parents/guardians. The program has the potential to support the establishment of healthy lifestyle habits from early childhood.

## Supporting information

S1 Appendix Questionnaire used in this study. (DOCX)
