# Supplementary material for: Development and implementation of an integrated oral health and nutrition education program using community children’s cafeterias: A pilot study
Source: PLoS One. 2026 Sep 3;21(9):e0357410. doi: 10.1371/journal.pone.0357410 (PMC13541141; doi:10.1371/journal.pone.0357410)
Supplement: Supplementary file 1 [file pone.0357410.s001.docx]

**Supporting information.**

**Supplementary Appendix 1. Questionnaire used in this study**

1. Please tell us your age. (____ years)
2. Please indicate your sex.
   Male / Female / Prefer not to answer
3. Please tell us your height and weight.
   (____ cm), (____ kg)
4. How many times do you brush your teeth per day?
   0 times / 1 time / 2 times / 3 or more times
5. I brush my teeth on my own without being told by someone else. (Children only)
   Every day / Once every 2 days / Once or twice a week / Rarely
6. I help my child brush their teeth (finishing brushing). (Parents/guardians only)
   Every day / Once every 2 days / Once or twice a week / Rarely
7. Do you observe the inside of your mouth?
   Every day / Once every 2 days / Once or twice a week / Rarely
8. Do you visit a dental clinic regularly?
   Yes / Not sure / No
9. Do you use fluoride products?
   Every day / Once every 2 days / Once or twice a week / Do not use / Do not know
10. How many meals do you eat per day?
    1 meal / 2 meals / 3 meals / 4 or more meals
11. Do you eat breakfast?
    Every day / Once every 2 days / Once or twice a week / Rarely
12. Do you think about nutritional balance when eating meals?
    Every day / Once every 2 days / Once or twice a week / Rarely
13. Do you count the number of times you chew when eating?
    Yes / Not sure / No
14. Do you leave hard foods uneaten?
    Yes / No

Do you eat foods that require chewing?
Every day / Once every 2 days / Once or twice a week / Rarely

15. Please tell us about your usual diet. Please circle the option that best describes how often you eat each of the following foods.

| Food item | Almost every day | Once every 2 days | Once or twice a week | Rarely |
| --- | --- | --- | --- | --- |
| Rice |  |  |  |  |
| Bread |  |  |  |  |
| Meat |  |  |  |  |
| Fish |  |  |  |  |
| Eggs |  |  |  |  |
| Milk and dairy products |  |  |  |  |
| Potatoes |  |  |  |  |
| Beans |  |  |  |  |
| Vegetables |  |  |  |  |
| Seaweeds |  |  |  |  |
| Fried foods |  |  |  |  |
| Fruits |  |  |  |  |
| Sweets |  |  |  |  |
